# Address on the Divine Mission of the Medical Profession

**Published:** 1889-04

**Authors:** H. K. Walker

**Affiliations:** Georgia


### Southern Medical Record.

A MONTHLY JOURNAL OF PRACTICAL MEDICINE.

All communications must be addressed to R. C. WORD, Managing Editor.

"*Quicquid Præcipies Esto Brevis.*"

VOL. XIX ATLANTA, GA., APRIL, 1889. NO. 4

### ORIGINAL AND SELECTED ARTICLES.

### ADDRESS ON THE DIVINE MISSION OF THE MEDICAL PROFESSION.

Delivered at the Southern Medical College Commencement, March 2nd, 1889 by the Rev. H. K. Walker, of Georgia.

Bobus Smith, the famous brother of the yet more famous clerical wit, Sidney Smith, was once discussing with Sir Henry Holland, the celebrated physician of London, the moral benefits conferred by the learned profession upon mankind. The discussion waxed warm, when Sir Henry as a clinching argument, tauntingly remarked that the "legal profession certainly did not make angels of men," whereupon Mr. Smith retorted, "very true, Sir Henry, very true, but the medical pro. fession certainly does."

I would be both too politic and too polite to introduce my address to this large and important assemblage of the "angel-making" fraternity with this time-worn jest at their expense, were it not that I intend to repudiate the covert sneer it contains, and transforming the sentiment from "jest to earnest," make it the *motto* for what I shall have to say this evening. The insinuation of Mr. Smith, that the medical profession succeeded admirably in robbing earth to people heaven, in this sense making angels of men, is the basest of base slanders. For while there are no doubt some isolated cases in which blundering diagnosis or mistaken treatment has expedited the passage of some traveler along life's rugged main, to that bourne from whence no traveler e'er returns, still these are exceptions, not the rule. Indeed, a more just complaint against the profession would be that they manage to keep down here on this earth a great many people who would no doubt be happier and far more useful in heaven. And it is a fact which I believe to be beyond gainsaying, that if the medical profession could have absolute and undisputed control of the physical en vironments, hygienic surroundings and moral habits of our race for one hundred years, making not only us, but themselves subject to the theoretical requirements of Medical Science, the average duration of human life would be doubled, earthly existence would be one long day of sunshine, and pain and suffering would be well nigh banished from the world. Of course such blissful possibilities can never come to pass, for no argument, not even the hope of long life and happiness can dissuade most men from committing involuntary suicide. Where poison and the pistol, war, famine and pestilence have slain their thousands, gluttony and drunkenness, licentiousness and laziness, filth and wantonness have slain their tens of thousands. Against these there seems to be no law, and in the presence of such deadly foes, the medical practitioner is many times practically powerless. In all seriousness, the odds against which every medical man, in his work of healing and restoration must battle are perfectly fearful, especially in this age of luxurious living and fashionable dissipation, when in addition to the hereditary diseases that have come down through the poisoned blood of bygone generations, a thousand other agencies are at work sapping the vitality, and undermining the constitution of the strongest and most enduring. Physical force and nerve power are ruthlessly sacrificed on the altars of business enterprise, social conventionalism and sensual enjoyment. Health is theoretically admitted to be the greatest of earthly blessings, but practically fame, fortune and rosy crowned pleasure are the coveted goals to gain which hours of physical anguish are willingly if not gladly borne. In spite of all these things, medical skill has advanced to so high a degree of perfection, that thousands of lives have been saved, which fifty, even twenty-five years ago, must have been lost, and the average duration of human life has gradually but constantly lengthened. This should be sufficient to prove that in the sense in which Bobus Smith meant it, physicians have long since ceased to be, if, indeed they ever were "*Angel makers.*

There is a time and proper sense however, in which this idle jest expresses, tho' in figurative language, the mission of medical men in this diseased and crippled old world of ours. It is a great mistake to suppose, that in this day and time, the duties and responsibilities of any class of men, are limited by professional routine or business engagements. Every man has an important work to do outside the special demands of his own calling. In one sense every man is called to God of be a minister of righteousness and a preacher of glad tidings. Especially is this true of the man who devotes himself to the study and practice of medicine. He is to do more than to alleviate the sufferings, repair the injuries, and preserve the life of his fellow men. This would seem to be work enough, but directly in line with these, there is work to be done in the sphere of morals, religious work to be done, for which he is specially fitted, and to which, I believe, the commanding voice of God as well as the pleading voice of humanity calls him.

In ancient times the disciples of Æsculapius were inducted into their office with solemn religious ceremonies, an oath of fealty to their profession, and of devotion to the interests of humanity bound them, and the blessing of the gods rested upon them. After what I have already said, it will occasion no surprise when I say that the ancients seemed to have a better and nobler conception of the true solemnity and dignity of the profession of medicine than we have to-day second only in rank to the sacred ministry, and hardly subordinate in importance when we consider the magnificent range of sublime possibilities that lie within its grasp, why should it be considered inappropriate if the most solemn religious rites and ceremonies should ratify and seal the entrance of these young men upon their noble life-work. Certainly they should derive large measures of peace and happiness from the thought that in practicing the art of healing, they are working out God's purposes, carrying out his Divine will and following in the footsteps of Christ, the great healer and Saviour of mankind. From all these considerations I draw the general propositions that *the medical profession has a divine mission*, that every physician is an angel of mercy and a messenger of spiritual as well as physical peace to his fellow-men.

It should be a source of infinite comfort and inspiration to you *young gentlemen* to feel and to know that you have been set apart to so grand a mission. Into every department of life you are fitted to enter. Your knowledge of the human frame, its vital phenomena, its organic laws, its thousand mysteries, enable you to minister to the sick and the suffering and the dying. Your very presence brings healing, your step brings quiet to throbbing nerves, and your smile a benediction to tired and fainting hearts. But your knowledge of mental physiology and of the terrible physical effects of vice and sin, fit you quite as thoroughly for activity and useful service in other spheres, as the purely physical, and it is my purpose to dwell rather upon this part of your mission to-night.

I esteem it not only a great honor, but a great privilege to speak upon this important subject to a class of students, whom I know are fitted by their previous training not only to appreciate but to act upon the suggestions that may be offered. If there be one distinguishing feature in the conduct of the Southern Medical College, that should more than any other commend it to the patronage and support of the public and especially the christian public, it is the inculcation of the highest code of morals in connection with medical instruction. With a faculty, the equal in intellectual ability of any in the South, if not in the nation, with an equipment and natural advantages surpassed by none, the school would have been worthy of and would doubtless have attained its *phenomenal success*, even if it had not emphasized the peculiar feature alluded to; and since the purest devotion to the cause of morals and religion has inspired the noble efforts in this direction, it would be eminently fitting for us to sing the long metre doxology, and on this her tenth anniversary, praise God from whom all blessings flow, for the past history and present prosperity of the Southern Medical College. Feeling confident, therefore, that I am not introducing an irrelevant or unfamiliar side issue to your attention, I go on to say that, I consider it the Divine mission of the medical profession to stand as a solid, immovable stone wall against the encroachments of modern materialism. I am perfectly aware that science and especially medical science, owes a vast debt of gratitude to the patient researches and painstaking investigations of men who are the champions of this gigantic "ism". I do not underrate the labors of that great anatomist, Prof. Thomas Huxley, who in his intense search into the hidden phenomena of second causes, has lost sight of the great first cause. I am not so narrow-minded but that I can heartily recognize the immense service to the World of science wrought by Charles Darwin, John Tyndall and Herbert Spencer. But with all the good that they have accomplished, there has come a vast amount of evil. Men are like sheep, they follow bell-wethers, and if the bell-wether jumps over a precipice they all come tumbling after. In consequence, this nineteenth century is an age in which materialism has flourished more than in any other age of the world's history. Just as a few generations ago there was a great rush toward rationalism, and a little later toward pantheistic deism, so to-day, there is a general setting in of the tide toward scientific materialism. Callow and inexperienced young men especially are embracing the new ism with great fervor, and becoming John Stuart Millites, Herbert Spencerites and Thomas Huxleyites, and alas that there should be so many good men, so many able men willing to lead them on and on, over the bleak and dreary deserts strewn with the skeletons of those who in bygone ages have wandered there only to perish. I need not tell you that medical students are specially tempted in this direction. Accustomed to study physical phenomena, delving deep down into the profoundest mysteries of human existence, it is no wonder that to their minds, matter is supreme. And not only this, but in the vast majority of our medical colleges the instruction given is clearly in favor of materialism, and in very many cases the professors are avowed materialists. No system of ethics is taught. Matter and motion are enthroned, mind and spirit are crucified. How easily then can young medical students be led astray. The wonder is that so large a proportion of them preserve their spiritual integrity and cleave to the faith of their fathers. And I am so thoroughly convinced of the tremendous risks involved and so profoundly impressed with the dangers to be confronted, that if I had a son desiring to study medicine, I would feel as tho' I were giving him a through ticket to Perdition should I buy him a ticket admitting him to the lectures delivered in many of our most prominent and finely equipped medical colleges. For this deadly poison is in the air, and to breathe it in means spiritual and eternal death. And by the same token, if I had a dozen sons whom I wanted to have a pure and sound medical education, I would send them all to the Southern Medical College, for I am satisfied from the lectures that I have heard and the addresses that I have read emanating from your distinguished professors, that if you young gentlemen have gone astray into the bleak and barren wilderness of materialism, you have yourselves and not your instructors to blame for it. And yet I am not ignorant of the fact that your very environment as a student of medicine, your experience in the dissecting room, when you carve deep down only to find no place in the dead body for the ever living soul, your patient search in the realm of biology, seeking to find out the origin of life, and above all your reading the works of men honored and revered in your profession, in which the claims of materialism are advanced with a force and complacency hard to withstand, all these things serve to weaken if not destroy your faith in the world of spirit, and make you if a foe at all, a friendly foe to the dreadful ism which robs this Universe of its God, and mankind of its Saviour.

It is because you know the mighty power of this enemy, because you have felt the force of its deadly assaults, that I appeal to you gentlemen of the medical profession to stand as a solid stone wall against it. Do not admit its foolish and fatal premises, no matter how wise and scientific and plausible they may seem. Combat its dangerous tendencies, brace yourselves against its insidious encroachments. Do this for your own sakes; your own happiness, your own salvation is at stake. But your mission is a far loftier one than that of self-defense. As a protection to the best interests of society, the medical profession should stand as an impregnable fortress against materialism.

Let me point out, very briefly, the way in which physicians may wield their mighty influence on the side of right and truth. When a young M. D. goes into any community with proper credentials and a sound moral character, he at once becomes a recognized factor in the life of that community. He may get little if any practice for the first few months, his professional visits may be purely imaginary, and he may spend most of his time riding around for amusement, taking his saddle bags or medicine case only for the sake of appearances, but at the same time he takes his position among the educated men of his neighborhood, and if he attends to his business, and does not spend two thirds of his time flirting with the girls, and trying to marry before he can make money enough to pay his own board bill, he will very soon attain considerable influence, especially over the young men with whom he comes in contact. Now, it must be candidly admitted, that in almost every community, no matter how rural or remote, there are some devotees more or less devout and aggressive, of the goddess of science, as opposed to revealed religion. Some empty pated young fellows think it very bright and smart to ventilate certain infidel views and opinions. For one thing, they ardently claim to be atheistic Evolutionists, they esteem it a great honor to be descended from apes and monkeys and babboons, and we can't help admiring their consistency, for they certainly do all they can to prove the allegation of their descent from such handsome and intelligent ancestors. If they find the young doctor intellectually inclined, they will either to test his intelligence or drive him insane, fling at him Herbert Spencer's definition, which to the average man is as "clear as mud," to the effect that "evolution is an integration of matter and a concomitant dissipation of motion during which the matter passes from an indefinite, incoherent homogeneity to a definite homogeneity, and during which the retained motion undergoes a parallel transformation." And if this does not enlighten him, they will dose him with seven-leagued words and seven inch ideas *ad nauseam*. Now if the young doctor has made good use of his time and opportunities, especially if he graduated from the Southern Medical College, he will not only be prepared to defend himself, but to carry the war into the enemy's camp. He will enlighten the youthful Spencerite by reminding him in the first place, that evolution is not necessarily atheistic. Whatever may be the individual opinions of christian men upon this threadbare subject, whatever may be their private views as to the ultimate outcome of the doctrine, as now taught, and by many thousands earnestly believed, all are agreed, that if the missing link is ever found and evolution scientifically demonstrated, there will be nothing found in it inconsistent with the purest theism. Some of the best and noblest and purest christians in the world to-day believe in the probable truth of evolutionary hypothesis. Moreover, the medical man can, with far greater effect than the minister of the Gospel, tell the persistent skeptic the difficulties that lie in his way—can demonstrate to him the utter absurdity of spontaneous generation, can expose the miserable subterfuges to which materialistic philosophers of the most resplendent genius have been compelled to resort, in order to explain the mental and spiritual phenomena by which we are continually surrounded, and show how persistently the facts of consciousness are disregarded, and above all, explain the awful results that would follow, if all men could be made to believe the wretched dogma. It would mean the loss of faith in a God; it would mean the orphanage of mankind, the funeral of our brightest, fairest hopes. No God, man without a soul, and destined very soon to perish forever, the race itself to be eventually blotted out; the earth itself, the sepulchre of all things else, to be consumed. No goal to attain; no grand final cause; only an aimless Universe. Who can fail to echo the mournful words of Strauss in speaking of this total eclipse of faith, when he said "The sense of utter abandonment is something awful. In the enormous machine of the Universe, amid the incessant whirl and hiss of its toothed iron wheels, amid the deafening crash of its ponderous stamps and hammers, in the midst of this whole terrific commotion, man, a helpless and defenceless creature, finds himself placed not secure for a moment, that on an imprudent motion on his part, a wheel may not seize him and rend him, or a hammer crush him to powder."

This in all its dreary baldness, is the creed of the Materialistic

Philosophy, and I contend that its mere statement is its sufficient refutation. But it may be asked why are medical men specially fitted for this crusade against materialism—why can not Ministers of the Gospel do all this work and leave physicians to the strictly legitimate exercise of their functions? Ah, yes, it is the same old story of the preacher pulling in the shafts, and the rest of the community, doctors, lawyers, merchants, mechanics, men, women children up in the wagon driving him to death. But to answer the question seriously, I would say that so far as doing the most effective service in this direction, the Minister's hands are completely tied. In this selfish age it has grown to be the custom for everybody either openly or secretly to suspect everybody else of being governed by self-interest. The consequence is that no matter how powerful and convincing the arguments of the Minister, no matter how clearly and pungently he presents the "evidence of things not seen"—the unspoken and often times the outspoken demurrer on the part of many who hear will be: "Oh well it is to his interest to have people believe that way. If they believed that this world was all—that there is no such thing as spirit, and that when men died that would be the last of them, then he would be like Othello, his occupation would be gone." And the neutralizing, damning effect of such an opinion as this can never be estimated on this side of Eternity. But in the case of the physician, all this is different. So far from having any selfish interest in upholding the doctrines of a spiritual kingdom, it might be supposed that his purely professional interest would be in the opposite direction. Certainly if materialism were the Universal Faith it would do the doctor's business no harm, and if mental and spiritual phenomena were everywhere regarded as simply the effect of diseased or morbid conditions of the physical brain, then he would certainly not have to compete with Faith Cure and Mind Cure, and christian Science Doctors any longer.

Relieved in this way of the odium of self-interest, and looked up to by the entire community as authority upon such subjects, I verily believe that the well equipped and conscientious Christian physician is the best fitted man in any community to deal with the problems of materialism. Put two young men in the same town, both consecrated to the work of the Divine Master, the one a Minister and the other a physician, and I believe that in this direction at least, the doctor will in five years accomplish ten-fold more good than the Minister. Nor is ther eany place in this whole country where such work is not sorely needed. In one of the smaller towns of this our own fair State, there is a young man well known for his infidel and materialistic opinions, who openly boasts that he has a larger crowd of young men in his office on Sunday morning to listen to him while he advocates his pet theories than are in any of the churches. This may be only an idle boast, but that materialism in some of its varied forms is robbing our churches of our brightest and ablest yonng men, must be admitted. The wild chase after riches and worldly preferment, the luxurious pampering of the body, and the starving of the soul, these are all so many symptoms of the dreadful disease which is devastating society, and casting a pall of awful darkness over the eternal Future that lies beyond. In the Divine Mission of the Medical Profession to overthrow this stalwart foe to human happiness, I have perfect confidence. And this work, mighty as it is, can be accomplished. Let medical men everywhere enter the lists of this Grand Crusade, and how soon would a revolution be wrought, and the sacred heart of Christ's blessed Religion be rescued from the rude grasp of the Monster Invader.

Will you bear with me while I mention in conclusion some of the considerations that should incite you to do the noble work that I have so imperfectly outlined.

In the first place, you will have the satisfaction of knowing that you are ministering not only to the bodies but the souls of your fellow creatures, and thus in the truest sense help to "make angels of men." The human part of your mission is indeed a grand and glorious one. To ease pain and deadly weariness, to enter into some quiet, darkened home, and there in a hand to hand conflict drive back the ruthless destroyer, Death—to go into palace and cottage and hovel, carrying balm and sunshine everywhere—to meet and conquer Pestilence—to smooth the dying pillow—this is work, glorious enough to fill the hearts and hands of angels. No wonder that we crown you as our Prince, our Hero. No wonder that the choicest benediction of Heazen is given to those to whom the Saviour is represented as saying on that great day of Final Judgment—I was sick and ye visited me. But how Transcendently glorious is this work when combined with that of helping and saving Souls. Nobody knows better than you, gentlemen of the Medical Profession, the utter fallacy of any explanation of the origin of Life that leaves out a Personal God. You know the mysteries of the human frame—the beautiful mechanism—the Divine Wisdom manifested in every part. You know too the brutalizing effects of materialistic philosophy, how grand it is then to study Nature and then point man up to Nature's God.

But the effect upon your own souls is no less elevating and inspiring. How hardened you will inevitably become if you fail to carry out what I believe to be your divine mission. You will develop into a man of iron or marble. Natural sympathies will be frozen at the fountain. You will learn to look on man's sufferings with but little more feeling than those of a brute soon to perish forever. But, young gentlemen, I am persuaded better things of you. Wherever you go take Christ the Great Healer as your model in all things, and in so doing you will not only help to make "Angels of your fellow men," but angels of yourselves, bright winged messengers of joy and peace. And even in this life you will get some bright glimpses of that spirit world, which I have called upon you to-night to uphold and defend, and bye and bye you will be crowned with immortal glory.

